# Serum concentrations of free fatty acids are associated with 3-month mortality in acute heart failure patients

**DOI:** 10.1515/cclm-2019-0037

**Published:** 2019-06-07

**Authors:** Vesna Degoricija, Matias Trbušić, Ines Potočnjak, Bojana Radulović, Gudrun Pregartner, Andrea Berghold, Hubert Scharnagl, Tatjana Stojakovic, Beate Tiran, Saša Frank

**Affiliations:** University of Zagreb School of Medicine, Zagreb, Croatia; and Department of Medicine, University Hospital Centre Sisters of Charity, Zagreb, Croatia; Department of Medicine, University Hospital Centre Sisters of Charity, Zagreb, Croatia; University Hospital Centre Zagreb, Zagreb, Croatia; Institute for Medical Informatics, Statistics and Documentation, Medical University of Graz, Graz, Austria; Clinical Institute of Medical and Chemical Laboratory Diagnostics, Medical University of Graz, Graz, Austria

**Keywords:** acute heart failure, free fatty acids, lipolysis, mortality

## Abstract

**Background:**

Plasma free fatty acids (FFA) are higher in heart failure (HF) patients compared to healthy controls. Considering that the extent of FFA elevation in HF might mirror the severity of HF, we hypothesized that the serum levels of FFA may be a useful prognostic indicator for 3-month mortality in acute heart failure (AHF).

**Methods:**

We analyzed the serum samples of AHF patients obtained at admission to the emergency department. Serum levels of FFA were analyzed using an enzymatic reagent on an automatic analyzer.

**Results:**

Out of 152 included AHF patients that were originally included, serum samples of 132 patients were available for the quantification of FFA. Of these, 35 (26.5%) died within 3 months of onset of AHF. These patients had significantly higher serum levels of FFA compared to AHF patients who were alive 3 months after onset of AHF. Univariable logistic regression analyses showed a significant positive association of FFA levels with 3-month mortality (odds ratio [OR] 2.76 [95% confidence interval 1.32–6.27], p = 0.010). Importantly, this association remained significant after adjusting for age and sex, as well as for further clinical and laboratory parameters that showed a significant association with 3-month mortality in the univariate analyses.

**Conclusions:**

We conclude that the admission serum levels of FFA are associated with 3-month mortality in AHF patients. Therefore, measurements of circulating FFA levels may help identifying high-risk AHF patients.

## Introduction

Heart failure (HF) is a final stage of various cardiovascular diseases and therefore a frequent cause of disability and death worldwide [1].

Hemodynamic impairment in HF triggers compensatory mechanisms causing neurohormonal and metabolic dysfunction, which contribute to the disease progression and severity [2]. Left ventricular dysfunction and heart congestion trigger a compensatory elevation of catecholamines and natriuretic peptides. By contrast, right-sided HF and the consequent peripheral venous congestion triggers a systemic inflammatory response [3, 4]. Increased serum levels of catecholamines, natriuretic peptides and inflammatory cytokines are the principal drivers of catabolic dominance, in particular adipose tissue lipolysis, the hallmark of metabolic dysfunction in HF [5, 6]. Free fatty acids (FFA) generated during lipolysis are transported to the liver, skeletal muscles and the heart, where they are used as an energy substrate. Consequently, the serum levels of FFA are determined by the rate of their generation and usage.

High concentrations of circulating FFA are potent inducers of insulin resistance and systemic metabolic impairment [7]. Elevated FFA have been shown to promote vascular oxidative stress and inflammatory response, both the known triggers and hallmark of endothelial dysfunction [8–10]. Together with an augmented myocardial FFA uptake and utilization as energy substrate, as well as excessive FFA storage in cardiomyocites, these may contribute to myocardial dysfunction [11, 12]. Plasma FFA have been found to be higher in HF patients compared to healthy controls [13] and raised in HF patients with reduced rather than preserved left ventricular ejection fraction (EF) [14].

If the extent of FFA elevation in HF mirrors the hemodynamic impairment, then we hypothesized that the serum levels of FFA in the patients’ serum at admission may have a prognostic value for mortality in AHF. The aim of the present study was therefore to evaluate the prognostic value of the serum levels of FFA for 3-month mortality in AHF patients.

## Materials and methods

### Study design and patients

This was a prospective, observational single-center study in AHF. All subjects gave their informed consent for inclusion prior to participation in the study which was conducted in compliance with the ethical guidelines of the Declaration of Helsinki [15]. Ethics Committee approvals were obtained from the University Hospital Centre Sisters of Charity, Zagreb, Croatia (EP-15389/13-4) and the Medical University of Graz, Austria (29–266 ex 16/17). Patients with AHF as a primary diagnosis were recruited from the Emergency Department between November 2013 and February 2015. The diagnosis of AHF was established according to the ESC and ACCF/AHA Guidelines for HF by time of onset, final clinical presentation and ejection fraction (EF) [16–18]. Left ventricular EF was automatically calculated using the Simpson method. Patients with severe renal failure (serum creatinine ≥400 μmol/L) renal replacement therapy, major systemic disease, severe hepatic cirrhosis (Child-Pugh Class B or C), neoplasms, acute or chronic inflammatory disease at admission, recent trauma or surgery, pregnancy or younger than 18 years were not included in the study. A further exclusion criterion was the patient’s choice not to participate in the study. One hundred and fifty-two included AHF patients were treated according to the ESC Guidelines for AHF [17, 18]. Hypertension was diagnosed according to the ESC criteria [19]. Mean arterial pressure (MAP) was calculated as (systolic + 2 · diastolic blood pressure)/3. Systolic pulmonary blood pressure (SPAP) was approximated by the tricuspid valve velocity, the estimated central vein pressure and the Bernoulli equation from a Doppler echocardiography. The New York Heart Association (NYHA) Functional Classification is estimated according to the severity of patients’ symptoms, and the differentiation of worsening vs. *de novo* cases was based on the patients’ history.

### Laboratory procedures

The collection of the blood samples, and the standard laboratory methods, have already been described in previous reports on our AHF cohort [20–23]. Blood from the patients was obtained on admission to the hospital. The fasting status of the included AHF patients was unknown. Routine laboratory analyses including serum creatinine, urea, total cholesterol, low-density lipoprotein (LDL) cholesterol, high-density lipoprotein (HDL) cholesterol, triglycerides, alanine aminotransferase (ALT), aspartate aminotransferase (AST), and C-reactive protein (CRP) were measured using a Beckman Coulter instrument AU 2700, 2007 (Brea, CA, USA) and Architect c8000, Abbott 2013 (Chicago, IL, USA). Serum levels of FFA were analyzed using an enzymatic reagent (ACS-ACOD method) from Wako Chemicals (Neuss, Germany) as described previously [24]. The measurements were performed on an Olympus AU640 automatic analyzer. Glomerular filtration rate (GFR) was estimated by the Chronic Kidney Disease Epidemiology Collaboration (CKD-EPI) formula [25]. Interleukin-6 (IL-6) serum levels were determined using a specific chemiluminescent ELISA (QuantiGlo; R&D Systems, Wiesbaden-Nordenstadt, Germany). An electrochemiluminescence immunoassay with Elecsys e411 (Roche Diagnostics GmbH, Mannheim, Germany) was used for the quantification of N-terminal pro-brain natriuretic peptide (NT-proBNP). Blood cell counts and hemoglobin were measured in full blood supplemented with K3EDTA by an automatic analyzer DxH (Beckman Coulter).

### Statistical analyses

FFA serum levels of patients who survived were compared with those who either died in the hospital or within 3 months after onset of AHF using the Mann-Whitney U-test. Correlations between FFA and various laboratory and clinical parameters were determined using Spearman’s correlation coefficient due to the skewed distribution of many of the laboratory parameters.

Univariable and multivariable logistic regression analyses were used to examine the impact of FFA on 3-month mortality. In the multivariable analyses, we adjusted for age and sex, as well as for further clinical and laboratory parameters that showed a significant association with 3-month mortality in the univariate analyses. Results are presented as odds ratio (OR) and the respective 95% CI. R version 3.4.4 was used for these analyses.

## Results

### Patients’ clinical characteristics and laboratory parameters

The baseline characteristics, comorbidities, medication, laboratory results and outcome of our 152 AHF patients have been described elsewhere [20–23]. The mean patient age was 75.2 ± 10.3 years, with a range of 45.5–96.7, and 79 (52%) were female. Serum samples of 132 patients were available for the analyses presented here. Of these, 35 (26.5%) died within 3 months of onset of AHF. The median and range for serum FFA levels were 0.87 (0.13–2.71) mmol/L. There was no difference between AHF patients with available FFA measurements and those without, with respect to baseline characteristics, laboratory parameters and comorbidities (Supplementary Tables S1–S3).

### Correlation of FFA concentrations with laboratory and clinical parameters

As shown in Table 1, serum levels of FFA were significantly positively correlated with NT-proBNP, ALT, AST and IL-6. However, the serum levels of FFA were not correlated with age, body mass index (BMI), MAP, glucose and several markers of kidney function. Furthermore, no significant correlation with the serum lipids was found, with the exception of HDL cholesterol, for which a significant negative correlation was observed.

### Serum levels of FFA in AHF patients who died within 3 months after onset of AHF were higher compared to those who survived

**Figure 1:**
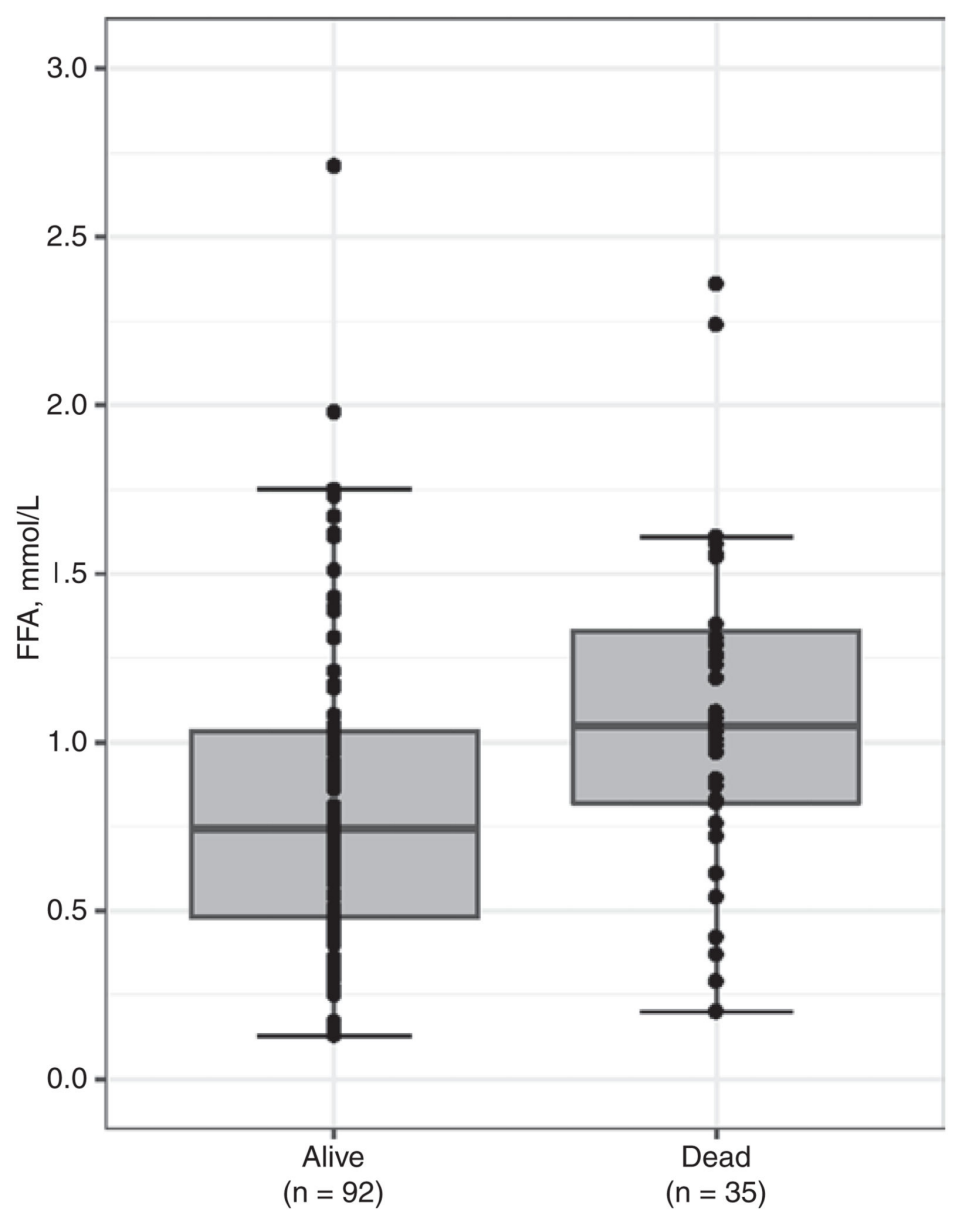
Serum levels of FFA in AHF patients. Difference between patients who died within 3 months after onset of AHF and those who survived.

Patients who died within 3 months after onset of AHF had significantly higher serum levels of FFA compared to AHF patients who were alive 3 months after onset of AHF (median and range: 0.74 [0.13–2.71] mmol/L vs. 1.05 [0.20–2.36] mmol/L, p = 0.004) (Figure 1).

### Logistic regression analyses

The univariable analyses showed that 3-month mortality was significantly positively associated with the serum levels of FFA, NT-proBNP, AST, ALT and IL-6, as well as significantly negatively associated with MAP, serum cholesterol levels and GFR (Table 2). Importantly, the association of the serum FFA levels with 3-month mortality remained significant after adjusting for age and sex as well as for the clinical and laboratory parameters which were significantly associated with 3-month mortality in the univariate analyses. Due to the high collinearity between AST and ALT, two different models were fit (Table 3).

## Discussion

This study is the first that shows the association of serum levels of FFA with 3-month mortality in AHF patients. After adjusting for variables that were both known to influence the prognosis in HF and significantly associated with 3-month mortality in the univariate analyses in the present study, the association of FFA with 3-month mortality remained significant.

It is known that a decreased cardiac output and a subsequently decreased tissue perfusion as well as venous volume overload and congestion are accompanied by increased serum levels of catecholamines, natriuretic peptides and inflammatory cytokines, which are potent inducers of adipose tissue lipolysis [5, 6, 26]. The positive correlations of serum FFA with NT-proBNP and IL-6 that were observed in the present study substantiate the relationship between serum levels of FFA, the rate of adipose tissue lipolysis, and the severity of AHF. Therefore, it is reasonable to infer that the higher levels of FFA in AHF patients who died are simply a consequence of the severe stage of the disease itself associated with higher levels of catecholamines and natriuretic peptides as well as a more pronounced inflammatory response and insulin resistance. However, the capacity of elevated circulating FFA to increase the activity of the sympathetic nervous system [27, 28] suggests that FFA, which were initially increased as a consequence of hemodynamic and metabolic disturbances in HF, contribute to exaggeration of these pathophysiologic processes. This, together with the established detrimental effects of elevated FFA on the cardiovascular system as well as their role in cardiac lipotoxicity [8–12], suggests that elevated FFA play an active role in the HF pathophysiology. Indeed, previous studies have shown that an increased uptake of FFA into cardiac myocytes, secondary to the elevated FFA serum levels, diminishes glucose utilization and deteriorates cardiac ATP production [29]. In line with this, drugs which interfere with FFA utilization for energy production and shift substrate utilization towards glucose have been shown to exert positive effects on cardiac function in patients with chronic HF [30, 31]. Considering this and higher serum FFA levels in AHF patients who died within 3 months after the onset of AHF compared to those who survived, as observed in the present study, it is tempting to assume that the pharmacological inhibition of FFA utilization might improve cardiac function and reduce mortality in AHF.

In the present analysis, serum levels of FFA were also positively correlated with ALT and AST, whose increased serum levels indicate hepatocyte damage. Hepatocyte damage in HF is primarily due to liver hypoperfusion caused by a decreased cardiac output and to a lesser extent due to passive hepatic congestion, a consequence of rightsided HF [32]. A healthy liver takes up substantial amounts of FFA that are liberated during lipolysis from the adipose tissue and uses them as fuel or substrate for ketone body synthesis. Compared to AHF patients who were alive, AST levels in the present study were significantly higher in AHF patients who died within 3 months after onset of AHF (median and range: 25.0 [10–666] U/L vs. 36.0 [14–487] U/L, p = 0.001). Accordingly, the increased FFA levels in AHF patients who died might at least in part be a consequence of the decreased FFA uptake by the liver, which was more damaged in patients who died compared to those that did not.

Additionally, the altered energy substrate preference of the failing heart, namely a shift from FFA to glucose [5, 33], may have been more pronounced in patients with a more severe disease. This may have contributed to the higher FFA serum levels in AHF patients who died within 3 months after onset of AHF compared to patients who survived.

There are several limitations to our present study: the design precludes drawing conclusions about cause and effect for the pathophysiological processes involved in the regulation of the serum levels of FFA. Moreover, we have no data on whether and how long patients were fasting before blood collection. Furthermore, due to missing values in the outcome as well as some of the regressor variables, data from only 117 patients was included in the multivariable regression model presented. The moderate number of available serum samples (n = 132) in this monocentric study influences the statistical power of our analyses. Therefore, larger studies are needed to confirm our results.

## Conclusions

Based on our results, we conclude that admission serum levels of FFA are associated with 3-month mortality in AHF patients. Therefore, measurements of circulating FFA levels might help identifying high risk AHF patients.

## Supplementary Material

The online version of this article offers supplementary material (https://doi.org/10.1515/cclm-2019-0037).

Supplementary Tables 1-3

## Figures and Tables

**Table 1:** Correlation analyses of FFA with clinical and laboratory parameters.

		FFA, mmol/L	
	
	r	p-Value	n
Age, years	0.08	0.360	132
BMI, kg/m^2^	–0.12	0.185	132
MAP, mmHg	–0.04	0.685	132
NT-proBNP, pg/mL	0.27	**0.002**	126
GFR, mL/min/1.73 m^2^	–0.04	0.690	131
Urea, mmol/L	0.14	0.111	131
Creatinine, mol/L	0.05	0.589	131
ALT, U/L	0.20	**0.021**	128
AST, U/L	0.24	**0.005**	129
IL-6, pg/mL	0.24	**0.005**	132
Total cholesterol, mmol/L	–0.17	0.057	132
LDL cholesterol, mmol/L	–0.13	0.126	132
HDL cholesterol, mmol/L	–0.18	**0.043**	132
Triglycerides, mmol/L	–0.05	0.542	132
Glucose, mmol/L	–0.03	0.697	127

Data presented are the Spearman correlation coefficient r, the corresponding p-value, and number of available samples (n); significant correlations are depicted in bold. ALT, alanine aminotransferase; AST, aspartate aminotransferase; BMI, body mass index; FFA, free fatty acids; GFR, glomerular filtration rate; HDL, high-density lipoprotein; IL-6, interleukin 6; LDL, low-density lipoprotein; MAP, mean arterial pressure; NT-proBNP, N-terminal pro brain natriuretic peptide.

**Table 2:** Univariable logistic regression analyses to assess the association of FFA and various clinical and laboratory parameters with 3-month mortality.

	OR (95% CI)	p-Value	Events/n
FFA, mmol/L	2.89 (1.30–6.79)	**0.010**	35/127
Age, years	1.02 (0.98–1.07)	0.302	35/127
Sex	1.52 (0.70–3.37)	0.296	35/127
BMI, kg/m^2^	0.95 (0.88–1.02)	0.185	35/127
NT-proBNP, ng/mL	1.05 (1.02–1.09)	**0.002**	34/121
GFR, mL/min/1.73 m^2^	0.97 (0.95–0.99)	**0.008**	34/126
MAP, mmHg	0.98 (0.96–1.00)	**0.030**	35/127
EF, %	0.97 (0.94–1.01)	0.161	27/118
Cholesterol, mmol/L	0.65 (0.40–0.99)	**0.029**	35/127
LDL cholesterol, mmol/L	0.47 (0.14–1.36)	0.054	35/127
HDL cholesterol, mmol/L	0.66 (0.45–0.94)	0.204	35/127
Log(triglycerides), mmol/L	0.58 (0.20–1.54)	0.291	35/127
Glucose, mmol/L	0.94 (0.84–1.02)	0.187	34/123
AST, U/L	1.01 (1.00–1.02)	**0.015**	34/125
ALT, U/L	1.01 (1.00–1.02)	**0.031**	34/124
IL-6, pg/mL	1.01 (1.00–1.01)	**0.030**	35/127
T2D	0.93 (0.43–2.05)	0.859	35/126
Smoking	0.53 (0.18–1.34)	0.202	35/127

Significant associations are depicted in bold. ALT, alanine aminotransferase; AST, aspartate aminotransferase; AHF, acute heart failure; CI, confidence interval; CRP, C-reactive protein; EF, ejection fraction; FFA, free fatty acids; GFR, glomerular filtration rate; HDL, high-density lipoprotein; IL-6, interleukin-6; LDL, low-density lipoprotein; MAP, mean arterial pressure; NT-proBNP, N-terminal pro brain natriuretic peptide; OR, odds ratio; T2D, type 2 diabetes.

**Table 3:** Multivariable logistic regression analyses of FFA and 3-month mortality.

	OR (95% CI)	p-Value	Events/n
Model 1	2.94 (1.12–8.28)	0.033	32/118
Model 2	2.77 (1.06–7.75)	0.042	32/117

Model 1 was adjusted for age, sex, NT-proBNP, GFR, MAP, cholesterol, AST, and IL-6. Model 2 was adjusted for age, sex, NT-proBNP, GFR, MAP, cholesterol, ALT, and IL-6. ALT, alanine aminotransferase; AST, aspartate aminotransferase; CI, confidence interval; FFA, free fatty acids; GFR, glomerular filtration rate; IL-6, interleukin-6; MAP, mean arterial pressure; NT-proBNP, N-terminal pro brain natriuretic peptide; OR, odds ratio.
