# Supplementary material for: Serum concentrations of free fatty acids are associated with 3-month mortality in acute heart failure patients
Source: Clin Chem Lab Med. Author manuscript; Available in PMC 2019 Oct 25. (PMC6779572; doi:10.1515/cclm-2019-0037)
Supplement: Supplementary Tables 1-3 [file EMS83439-supplement-Supplementary_Tables_1_3.pdf]

Supplemental Table 1. Differences in baseline characteristics and pre-admission medication between AHF patients that were and those that were not analyzed in the present study

	Analyzed (N=132)	Not analyzed (N=20)	p-value
Age (years)	77.3 (45.5-92.4)	77.6 (55.6-96.7)	0.779
Female	65 (49.2%)	14 (70%)	0.097
BMI (kg/m^2)	28.4 (16.3-43.5)	31.0 (21.3-42.0)	0.401
Weight (kg)	80.0 (40.0-144.0)	81.5 (60.0-130.0)	0.741
Smoker	34 (25.8 %)	4 (20%)	0.783
NYHA class			0.484
2	11 (8.3%)	0 (0%)	
3	72 (54.4%)	11 (55%)	
4	49 (37.1%)	9 (45%)	
MAP (mmHg)	103.3 (53.3-160.0)	103.3 (73.3-130.0)	0.576
Heart rate (beats/min)	101.0 (36.0-160.0)	98.5 (50.0-150.0)	0.372
EF (%)	40.0 (20.0-70.0)	52.0 (20.0-70.0)	0.052
SPAP (mmHg)	45.0 (35.0-80.0)	45.0 (40.0-70.0)	0.896
JVD	48 (34.8%)	7 (35%)	0.207
Enlarged liver	46 (34.8%)	7 (35%)	1.000
Peripheral edema	93 (70.5%)	12 (60%)	0.436
Ascites	21 (15.9%)	0 (0%)	0.077
Statins	35 (26.5 %)	6 (30%)	0.789
ß-blockers	55 (42.6%)	12 (60%)	0.157
ACEI	73 (55.3%)	13 (68.4)	0.329
Amplodipine	21 (16.0%)	4 (20%)	0.746
AHF type			0.796
Worsening of CHF	92 (69.7%)	13 (65%)	
De novo AHF	40 (30.0%)	7 (35%)	

Data are presented as n (%) or as median and range (minimum to maximum). Differences between AHF patients who were and those who were not analysed in the present study were tested using Fisher's exact test or the Mann-Whitney U test.

AHF, acute heart failure; ACEI, angiotensinogen converting enzyme inhibitor; BMI, body mass index; EF, ejection fraction; JVD, jugular venous distension; MAP, mean arterial pressure; NYHA, New York Heart Association Functional Classification; SPAP, systolic pulmonary artery pressure.

Supplemental Table 2. Differences in laboratory parameters between AHF patients that were and those that were not analyzed in the present study

	Analyzed (N=132)	Not analyzed (N=20)	p-value
GFR (ml/min/1.73 m^2)	51.7 (15.0-105.7)	42.6 (20.0-101.1)	0.226
Urea (mmol/L)	8.0 (3.0-64.0)	11.5 (4.0-41.0)	0.581
Creatinine (µmol/L)	104.0 (53.0-273.0)	127.0 (69.0-255.0)	0.635
NT-proBNP (ng/mL)	9.60 (0.2-70.0)	7.8 (0.8-35.0)	0.904
ALT (U/L)	23.0 (6.0-556.0)	25.0 (12.0-623.0)	0.230
AST (U/L)	27.0 (10.0-487.0)	30.0 (13.0-666.0)	0.242
Serum protein (g/L)	68.0 (31.0-87.0)	69.5 (36.0-80.0)	0.450
Albumin (g/L)	40.0 (21.0-62.0)	38.5 (29.0-72.0)	0.804
IL-6 (pg/mL)	19.4 (0.4-300.0)	30.6 (3.1-153.9)	0.301
CRP (µg/mL)	9.7 (0.2-247.4)	7.4 (1.9-84.0)	0.689
Total cholesterol (mmol/L)	3.9 (1.7-8.5)	3.7 (2.2-9.1)	0.442
LDL cholesterol (mmol/L)	2.3 (1.0-6.1)	2.1 (0.8-6.3)	0.318
HDL cholesterol (mmol/L)	1.0 (0.3-3.6)	1.0 (0.4-1.9)	0.788
Triglycerides (mmol/L)	1.0 (0.5-4.3)	1.2 (0.7-2.8)	0.362

Data are presented as median and range (minimum to maximum). Differences between AHF patients who were and those who were not analysed in the present study were tested with the Mann-Whitney U test.

ALT, alanine aminotransferase; AST, aspartate aminotransferase; AHF, acute heart failure; CRP, C-reactive protein; GFR, glomerular filtration rate; HDL, high-density lipoprotein; IL-6, interleukin-6; LDL, low-density lipoprotein; NT-proBNP, N-terminal pro brain natriuretic peptide;

Supplemental Table 3. Differences in the incidence of comorbidities between AHF patients that were and those that were not analyzed in the present study

	Analyzed (N=132)	Not analyzed (N=20)	p-value
Hypertriglyceridemia	52 (39.4%)	8 (40.0%)	1.000
Hypercholesterolemia	51 (38.6%)	8 (40%)	1.000
Hypertension	118 (89.4%)	18 (90.0%)	1.000
Hyponatremia	24 (18.2%)	2 (10.0%)	0.529
Anemia	35 (26.5%)	4 (20.0%)	0.784
T2D	71 (54.2%)	7 (35.0%)	0.149
COPD	35 (26.5%)	6 (30.0%)	0.789
CKD	41 (31.1%)	9 (45.0%)	0.306
CM	98 (74.2%)	15 (75.0%)	1.000
ACS	21 (15.9%)	3 (15.0%)	1.000

Data are presented as n (%). Differences between AHF patients who were and those who were not analysed in the present study were tested with Mann-Whitney U test.

Five patients were with unknown status regarding 3-month survival.

ACS, acute coronary syndrome; AHF, acute heart failure; CKD, chronic kidney disease; CM, cardiomyopathy; COPD, chronic obstructive pulmonary disease; T2D, type 2 diabetes.
